# Identification of *trkH*, Encoding a Potassium Uptake Protein Required for *Francisella tularensis* Systemic Dissemination in Mice

**DOI:** 10.1371/journal.pone.0008966

**Published:** 2010-01-29

**Authors:** Khaled Alkhuder, Karin L. Meibom, Iharilalao Dubail, Marion Dupuis, Alain Charbit

**Affiliations:** 1 Université Paris Descartes, Faculté de Médecine Necker-Enfants Malades, Paris, France; 2 INSERM, U1002, Unité de Pathogénie des Infections Systémiques, Paris, France; Cairo University, Egypt

## Abstract

*Francisella tularensis* is a highly infectious bacterium causing the zoonotic disease tularaemia. During its infectious cycle, *F. tularensis* is not only exposed to the intracellular environment of macrophages but also resides transiently in extracellular compartments, in particular during its systemic dissemination. The screening of a bank of *F. tularensis* LVS transposon insertion mutants on chemically defined medium (CDM) led us to identify a gene, designated *trkH*, encoding a homolog of the potassium uptake permease TrkH. Inactivation of *trkH* impaired bacterial growth in CDM. Normal growth of the mutant was only restored when CDM was supplemented with potassium at high concentration. Strikingly, although not required for intracellular survival in cell culture models, TrkH appeared to be essential for bacterial virulence in the mouse. In vivo kinetics of bacterial dissemination revealed a severe defect of multiplication of the *trkH* mutant in the blood of infected animals. The *trkH* mutant also showed impaired growth in blood ex vivo. Genome sequence analyses suggest that the Trk system constitutes the unique functional active potassium transporter in both *tularensis* and *holarctica* subspecies. Hence, the impaired survival of the *trkH* mutant in vivo is likely to be due to its inability to survive in the low potassium environment (1–5 mM range) of the blood. This work unravels thus the importance of potassium acquisition in the extracellular phase of the *F. tularensis* infectious cycle. More generally, potassium could constitute an important mineral nutrient involved in other diseases linked to systemic dissemination of bacterial pathogens.

## Introduction

*Francisella tularensis* is a Gram-negative bacterium responsible for the disease tularemia in a large number of mammalian species. Four different subspecies (subsp.) of *F. tularensis* that differ in virulence and geographic distribution have been characterized and are designated subsp. *tularensis* (type A), *holarctica* (type B), *novicida* and *mediasiatica*. *F. tularensis* subsp. *tularensis* is the most virulent subspecies causing a severe disease in humans, whereas *F. tularensis* subsp. *holarctica* causes a similar disease but of less severity [1]. Because of its high infectivity and lethality, *F. tularensis* is considered a potential bioterrorism agent [2].

*F. tularensis* is a facultative intracellular bacterium that infects and replicates mainly inside macrophages [3]. The molecular mechanisms by which *Francisella* adapts to life inside host cells has just begun to be elucidated. Many novel genes necessary for *Francisella* pathogenicity have been discovered in the past few years [4]. These include notably genes located in the *Francisella* pathogenicity island (FPI) [5], [6], [7], [8], [9], [10], [11], [12], and genes encoding the regulatory proteins MglA, SspA, FevR, PmrA and MigR, which regulate expression of the FPI [5], [13], [14], [15], [16], [17]; see [18] for a recent review. In addition, several recent genome-scale random and site-directed mutagenesis studies have led to the identification of novel genes important for replication inside macrophages and/or survival in mice [7], [19], [20], [21], [22], [23], [24]. However, the molecular mechanisms underlying the contribution of the identified genes to virulence have been addressed for only a limited number of them.

Remarkably, during its infectious cycle, *F. tularensis* is not only exposed to the intracellular environment of macrophages, but also to extracellular compartments [25], in particular to blood during its systemic dissemination [26], [27]. *F. tularensis* survival and multiplication in an infected host requires, in addition to the development of sophisticated strategies to subvert the host immune defences [28], [29], the capacity to acquire enough essential nutrients in each of the infected niches. We have recently shown that *F. tularensis* was able to use the available pool of intracellular gluthatione as a source of cysteine to multiply efficiently in eukaryotic host cells [24]. Since the availability of organic or mineral sources can vary considerably between the intracellular and the extracellular milieu, adaptation to these variations is crucial for *F. tularensis*. In this respect, several studies have already highlighted the importance of amino acid and nucleotide biosynthesis and uptake for the survival of pathogenic bacteria in blood (see for example [30] and references therein). However, bacterial adaptation to the peculiar ionic conditions encountered in blood has been largely understudied so far.

In the present study, the screening of a bank of LVS transposon insertion mutants on chemically defined medium led us to identify a mutant in a gene encoding the predicted potassium permease TrkH of the Trk uptake system. Confirming the importance of TrkH in potassium acquisition, normal growth of the *trkH* mutant in broth could only be restored by adding high potassium concentration. Strikingly, although not required for intracellular survival of LVS in cell culture models, TrkH appeared to play a major role in persistence and multiplication in the blood of infected mice.

## Results

### Phenotypic Screen for Auxotrophic Mutants

Our initial aim was to identify novel nutritional requirements of *F. tularensis*. Therefore, we used a simple screening procedure [31] to isolate putative auxotrophic mutants of *F. tularensis* LVS. Briefly, banks of Km^R^ colonies, generated by *HimarFT* transposon mutagenesis, were screened for growth on chemically defined medium (CDM). From approximately 2,500 clones, eight isolates failed to grow on CDM (*i.e.* 0.3%). This percentage is in agreement with the generally obtained values for that type of screens. The transposon insertion sites of the selected mutants were then determined (**Table 1**). Two mutants corresponded to a transposon insertion in genes of the purine biosynthetic pathway: one, in gene *FTL_1860* (or *purL,* encoding a phosphoribosyl formylglycinamidine synthase); and the other, in the adjacent gene *FTL_1861* (or *purF,* encoding an amidophosphoribosyl transferase). One mutant (*FTL_0028*) had an insertion upstream of a pyrimidine biosynthetic gene (132 bp upstream of *pyrB*, encoding an aspartate carbamoyltransferase). Two mutants in the aromatic amino acid biosynthetic pathway were identified; one in the gene *FTL_1966* (*trpE*, encoding an anthranilate synthase component I), and the other in *FTL_0173, (aroE,* a Shikimate 5-dehydrogenase). In addition to these auxotrophic mutants, one mutant had an insertion in gene *FTL_1962* encoding a hypothetical 113 amino acid long protein and one mutant had an insertion immediately upstream (11 bp) of gene *FTL_0699,* an ortholog of *rluD* of *E. coli* encoding the ribosomal large subunit pseudouridine synthase D. In *E. coli*, although the *rluD* gene is dispensable for growth, a *rluD* knock-out mutant is extremely slow-growing, suggesting that the *FTL_0699* mutant of LVS was selected on CDM because of a growth defect. Finally, two mutants corresponded to two distinct transposon insertions in gene *FTL_1708* (the first mutant carried an insertion 216 bp and the second one, 816 bp, after the start codon of *trkH*). The gene *FTL_1708* encodes a protein of 484 amino acids, homologous to the TrkH potassium transporters of *Salmonella typhimurium* and *E. coli* K12 (43% and amino acid identity and 65% amino acid similarity), and was hence designated *trkH*. Southern blot analysis confirmed the presence of a single transposon insertion in all the strains tested (**Figure S1**).

**Table 1:** Summary of mutants identified in screen.

Locus	Genomic location	Gene	Gene product	Cellular role^a^
FTL_1708	1,640,202	*trkH*	Potassium uptake protein	Environmental Information Processing; Membrane Transport
FTL_1708	1,640,802	*trkH*	Potassium uptake protein	Environmental Information Processing; Membrane Transport
FTL_1966	1,892,759	*trpE*	Anthranilate synthase component I	Metabolism; Amino Acid Metabolism
FTL_0173	177,040	*aroE*	Shikimate 5-dehydrogenase	Metabolism; Amino Acid Metabolism
FTL_1860	1,791,836	*purL*	Phosphoribosylformylglycinamidine synthase	Metabolism; Nucleotide Metabolism
FTL_1861	1,792,631	*purF*	Amidophosphoribosyltransferase	Metabolism; Nucleotide Metabolism
FTL_1962	1,889,732		Hypothetical protein	Unknown
FTL_0699^b^	687,717	*rluD*	Ribosomal large subunit pseudouridine synthase D	Genetic Information Processing; Translation
FTL_0028^b^	27,424	*pyrB*	Aspartate carbamoyltransferase	Metabolism; Amino Acid and Nucleotide Metabolism

a according to the KEGG database (http://www.genome.jp/kegg-bin/show_organism?org=ftl).

b insertion found in upstream region (11 bp upstream of *rluD* ; 132 bp upstream of *pyrB*).

Since the *trkH* gene had never been identified in previous screens, we hereafter decided to focus on the *trkH* mutant, and to evaluate the importance of potassium acquisition in *F. tularensis* physiopathology.

### In Silico Analysis

**Figure 1:**
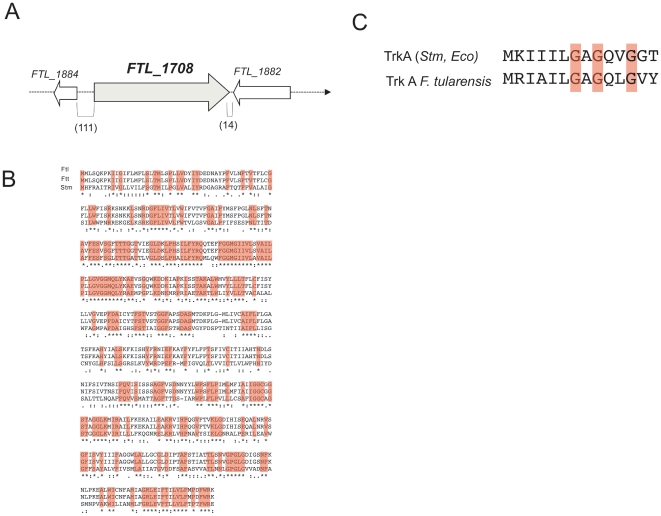
Genetic organzation of the *trkH* region and multiple alignments of the TrkH, TrkA proteins. (A) The gene *FTL_1708* (*trkH*) is flanked by two genes in opposite orientation. Parenthetic numbers give the sizes (in base pairs) of the intergenic regions flanking *FTL_1708*. (B) Alignment of the TrkH proteins of *F. tularensis* strains LVS (designated Ftl), Schu S4 (designated ftu) and TrkH of *S. typhimurium* LT2 (designated Stm) were performed using the Clustal W program. Residues that are identical in all strains (boxed in pink) are marked by “*”, conserved substitutions by a “:”, and semi-conserved substitutions by a “.” (C) Alignment of the N-terminal end of TrkA (from *E. coli* K12 and *S. typhimurium* LT2, upper line) and *F. tularensis* (from Schu S4 and LVS, lower line) encompassing the characteristic nucleotide binding motif GXGXXG at the N-terminus of TrkA. Pink residues are the conserved glycines involved in nucleotide binding.

The bacterial Trk potassium transport system consists of a membrane-spanning protein (TrkH), constituting the translocating subunit, and a peripheral membrane-associated nucleotide binding protein (TrkA), required to energize the system [32]. The genes encoding these two components are present and highly conserved in the genomes of the four *F. tularensis* subsps. The TrkH protein of *F. tularensis* LVS (encoded by *FTL_1708*) consists of 484 amino acids and is predicted to be a membrane protein with ten transmembrane helices (using the TMHMM prediction program). FTL_1708 shares 100% and 99.8% amino acid identity with TrkH of *F. tularensis* subsp. *holarctica* (OSU18 strain, FTH_1645), and *F. tularensis* subsp. *tularensis* (Schu S4 strain, FTT1638), respectively. The gene *FTL_1708* is flanked by two genes that are encoded on the opposite DNA strand, indicating that *FTL_1708* is transcribed as a monocistronic unit (**Figure 1A, B**). No paralog of *trkH* was identified in the LVS genome.

The *trkA* gene is not located in the same locus as *trkH* in the LVS chromosome, unlike in the *Vibrio vulnificus* genome [33] where the two genes are contiguous and constitute an operon. The LVS *trkA* gene (*FTL_1232*) encodes a protein of 457 amino acids sharing 29.2% identity (and 55.3% similarity) with TrkA of *E. coli* and SapG of *S. typhimurium* [34]. Of note, the N-terminus of FTL_1232 bears the characteristic nucleotide-binding motif GXGXXG found in TrkA porteins (**Figure 1C**).

**Figure 2:**
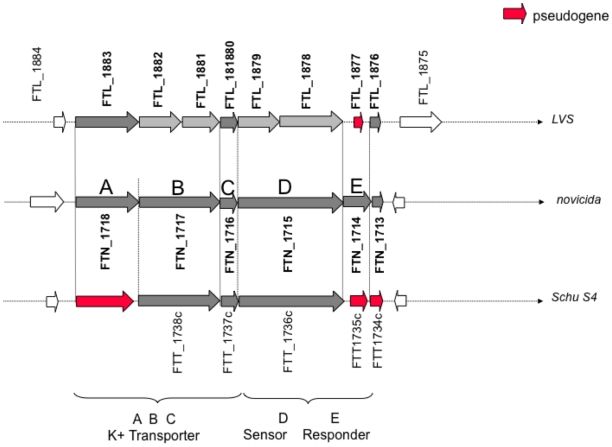
The *kdp* locus in *F. tularensis* subsp. *holartica* (LVS), *novicida*, and *tularensis*. The prototypical Kdp potassium transport system comprises: i) the transporter, composed of KdpA, KdpB and KdpC; and ii) the TCS, regulating its expression, and composed of KdpD, the sensor kinase; and KdpE, the response regulator. Upper line, the LVS locus. Whenever an FTL number has been attributed, it is indicated above the *orf*. Middle line, *F. tularensis* subsp. *novicida* which comprises an intact *kdp* locus. A, B, D and E, above each gene refer to *kdpA*, *kdpB*, *kdpC*, *kdpD* and *kdpE*, respectively. Lower line, *F. tularensis* subsp. *tularensis*. In dark grey, the intact genes; in light grey, the interrupted genes (but annotated as genes in the KEGG database); in red, the interrupted genes annotated as pseudogenes (in KEGG). **KdpB**. In *F. tularensis* subsp. *novicida*, the *kdpB* gene (*FTN_1717*; 2,040 bp) encodes a 679 aas protein. In LVS, the proximal portion of the gene carries: i) an in frame 63 bp deletion (deletion of nucleotides 509 to 571); and ii) a single nucleotide insertion (between nucleotides 1,096 and 1,097; *FTN_1717* numbering), leading to a premature termination of the coding sequence (creation of a TGA stop codon 9 bp downstream of the insertion). The resulting 374 aas truncated protein is designated *FTL_1882*. An ATG codon (in frame with the rest of the *kdpB* sequence of *FTN_1717)* is found immediately downstream of the stop codon (29 bp) of *FTL_1882*, leading to the prediction of a second open reading frame of 301 aas designated *FTL_1881*. Blastn analysis of the nucleotide sequence of LVS corresponding to gene *FTN_1717* (*i.e.* 1,978 bp from the start codon of *FTL_1882* to the stop codon of *FTL_1881*) reveals that the entire LVS sequence is 100% identical to *F. tularensis* subsp. *holarctica* FTNF002-00 genome region 1,811,308 to 1,809,331 and >99% identical to *F. tularensis* subsp. *holarctica* OSU18 genome region 1,815,047 to 1,813,070 (with a unique C to A substitution at position 71 of *FTL_1882*). Thus, in the three subsp. *holarctica* genomes available, the proximal part of the *kdpB* gene carries the same in frame deletion and the gene is interrupted by the same single nucleotide insertion. **KdpD**. In *F. tularensis* subsp. *novicida*, the *kdpD* gene (*FTN_1715*; 2,682 bp) encodes a 893 aas protein. In LVS, the corresponding gene is interrupted by a single nucleotide insertion (between nucleotides 981 and 982; *FTN_1715* numbering), leading to a premature termination of the coding sequence (creation of a TGA stop codon 32 bp downstream of the insertion). The resulting truncated predicted orf is designated *FTL_1879* (337 aa). An ATG codon (in frame with the rest of the *kdpD* sequence of *FTN_1715)* is found downstream of the stop codon (92 bp) of *FTL_1879*, leading to the prediction of a second orf of 525 aas, designated *FTL_1878*. Blastn analysis of the nucleotide sequence of LVS (2,683 bp) corresponding to gene *FTN_1715* reveals that the entire LVS sequence is 100% identical to *F. tularensis* subsp. *holarctica* OSU18 genome region 1,812,395 to 1,809,713100 and 99% identical to *F. tularensis* subsp. *holarctica* FTNF002-00 genome region 1,808,656 to 1,805,974 (with 3 single nucleotide substitutions). Thus, in the three subsp. *holarctica* genomes available, the *kdpD* gene is interrupted by the same single nucleotide insertion. **KdpE**. In *F. tularensis* subsp. *novicida*, the *kdpE* gene (*FTN_1714*; 687 bp) encodes a 228 aas protein. In LVS, the corresponding region is not predicted to encode a protein (*FTL_1877* is designated pseudogene). Comparison of the *F. tularensis* subsp. *novicida* and LVS nucleotide sequences reveal the presence of a 13 bp deletion in the proximal portion of the LVS sequence (deletion of nucleotides 45 to 57, *FTN_1714* numbering), resulting in a frame shift and the premature termination of the protein sequence (a TGA stop codon is found 11 bp downstream of the deletion). Blastn analysis of the nucleotide sequence of LVS (671 bp) shows a 100% identity with the *F. tularensis* subsp. *holarctica* OSU18 genome region 1,809,671 to 1,809,001; and >99% identity with *F. tularensis* subsp. *holarctica* FTNF002-00 genome region 1,805,932 to 1,805,262 (1 single nucleotide substitution) as well as with *F. tularensis* subsp. *tularensis* Schu S4 genome region 1,823,954 to 1,823, 284 (2 single nucleotide substitution). Thus, in the three subsp. *holarctica* genomes available and in Schu S4, the *kdpE* gene is a pseudogene resulting from the same 13 bp deletion. The regions containing the mutations in genes *kdpB* (*FTL_1881-1882*), *kdpD* (*FTL_1878-1879*) and *kdpE* (*FTL_1877*), have been resequenced by sequencing of cloned PCR products. This analysis fully confirmed the published sequence of LVS.

Genomic analyses suggest that *F. tularensis* subsp. *tularensis* and *holarctica* possess a single potassium transporter, a Trk-like transporter encoded by *trkH* and *trkA*. Indeed, the LVS and Schu S4 genomes presumably encode an inactive *kdpABC* potassium uptake system due to mutations in the *kdpB* and *kdpA* gene, respectively (**Figure 2**). Moreover, in both subspecies, the *kdpD/E* two-component system required to regulate *kdpABC* expression is also non-functional. In LVS, both the *kdpD* and *kdpE* genes harbors mutation; and Schu S4 possesses only an intact *kdpD* gene. In addition, no ortholog of the Kup potassium uptake system (also designated TrkD) could be identified in any of the four *F. tularensis* subsps. Hence, in both the LVS and Schu S4 strains, Trk is likely to be the unique active potassium uptake system.

### Growth Properties of the *trkH* Mutant in CDM

**Figure 3:**
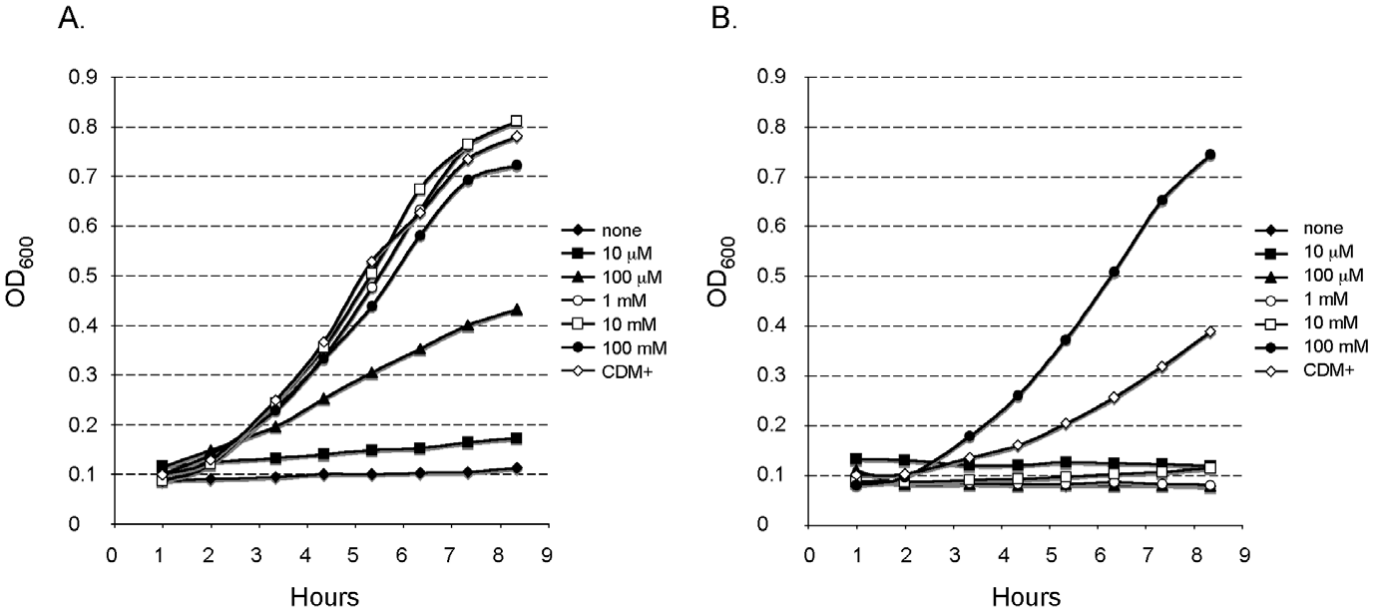
Growth properties of the *trkH* mutant in chemically definded medium. LVS (A) and *trkH* (B) were grown in chemically defined medium (CDM+) or in CDMx buffered with sodium phosphate and supplemented with KCl as potassium source (at concentrations indicated in legend).

We chose for all subsequent functional studies the *trkH* mutant strain carrying an insertion after the 216 bp of *trkH*. We first examined the impact of *trkH* inactivation on the ability of LVS to survive under low potassium conditions in broth (**Figure 3**). For this, LVS and *trkH* mutant strains were cultivated in normal chemically defined medium (CDM+) [35] or in CDM in which sodium phosphate had replaced potassium phosphate as the buffering agent (CDMx) and further supplemented with various concentrations of potassium chloride (KCl) as a source of potassium (ranging from 0 to 100 mM). Cultures were incubated at 37°C and growth was monitored over a 8 h-period (**Figure 3A, B**). The LVS strain grew normally in CDM+ medium while no growth was observed in CDMx devoid of potassium. The addition of only 10 µM potassium was sufficient to restore partial growth of LVS and normal growth was observed at and above 1 mM (*ie*. identical to that in CDM+, whose potassium concentration is approximately 18 mM). In contrast, the *trkH* mutant failed to grow at potassium concentrations at and below 10 mM. Growth comparable to wild type LVS was only restored at 100 mM potassium. Notably, partial growth of the *trkH* mutant was observed in CDM+, while on solid CDM+ medium, growth of the mutant was totally impaired.

**Figure 4:**
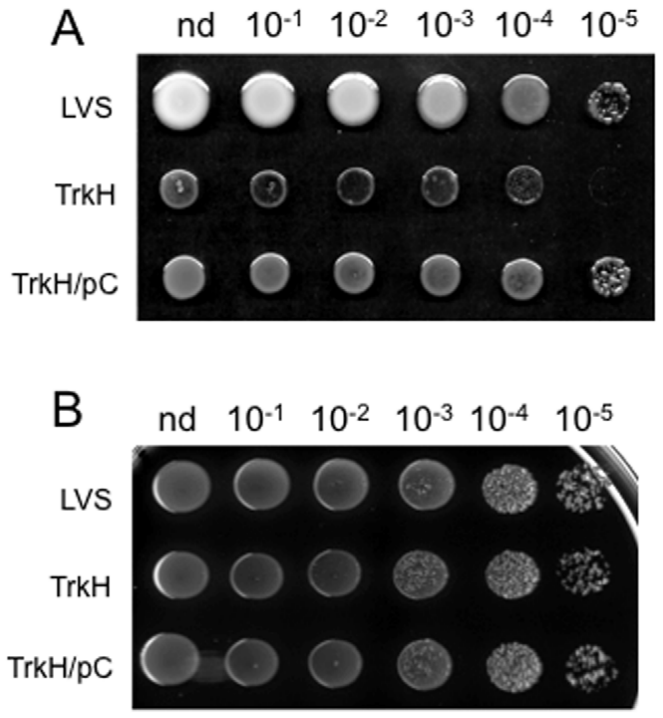
Functional complementation. Bacteria were grown in Schaedler K3 until an OD_600_ of 0.3. Ten µl of each culture (nd: non diluted, corresponding to *ca*. 10^7^ b) and of serial ten-fold dilutions, were spotted onto: CDM agar plate (A) and Chocolate agar plate (B). LVS: wild-type strain; TrkH: the *trkH* mutant derivative; TrkH/pC: the *trkH* mutant transformed with plasmid pC-*trkH* (a pFNLTP6 derivative carrying a wild-type *trkH* allele under control of the *gro* promoter).

We confirmed that the growth defect observed on CDM with the *trkH* mutant strain was due to the specific inactivation of *FTL_1708* by performing a functional complementation of the gene. For this, the recombinant plasmid pC-*trkH* carrying the wild-type *trkH* allele was introduced into the *trkH* mutant strain. On chocolate agar, growth of the mutant and complemented strains was indistinguishable from that of wild-type LVS. In CDM+ plates, growth of the *trkH* mutant was severely reduced and introduction of the recombinant plasmid pC-*trkH* restored normal growth (**Figure 4**). The apparent morphological differences between the growth of the WT and the complemented strains on CDM most likely reflects the faster growth of LVS on CDM solid medium.

These data confirm the absolute potassium requirement for LVS growth. Furthermore, supporting the in silico analyses, the properties of the *trkH* mutant strongly suggest that TrkH is the major potassium transporter of LVS.

### Sensitivity to Serum and to Osmotic Shock

**Figure 5:**
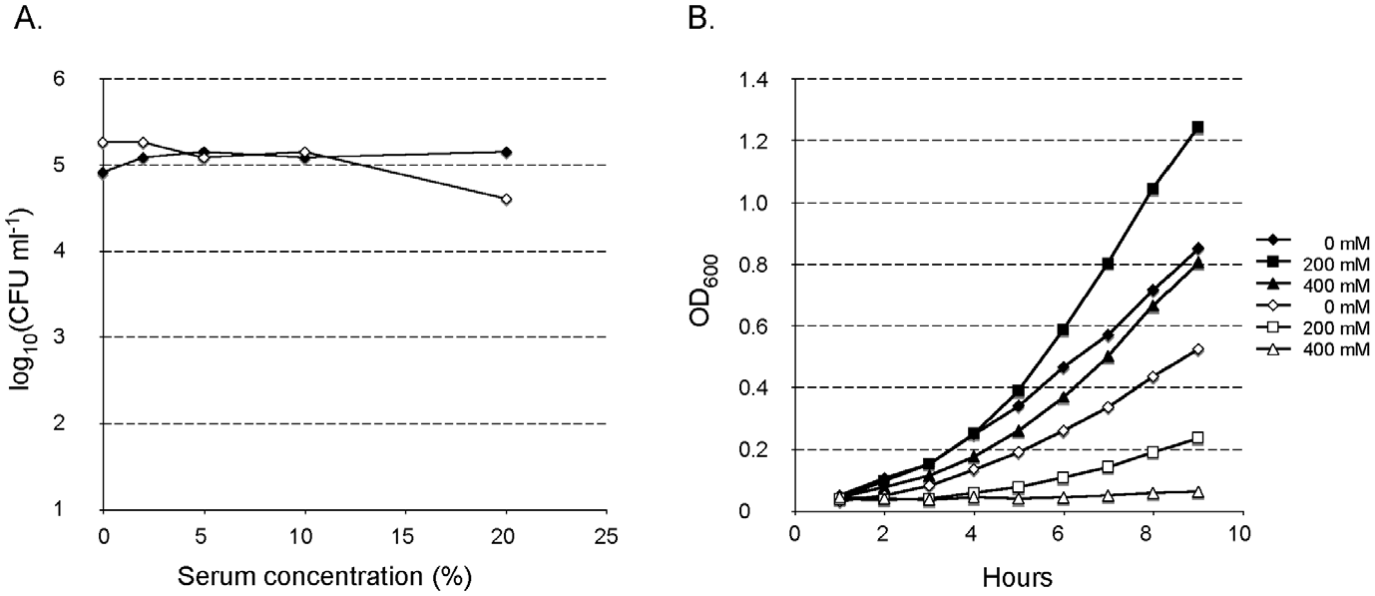
Resistance to serum and high osmolarity. Survival of LVS (closed symbols) and *trkH* mutant (open symbols) after 1 hour incubation in human non-decomplemented serum (0–20%) (A). Growth of LVS (closed symbols) and *trkH* (open symbols) in CDM supplemented with NaCl as indicated in legend (B).

The Trk system of *V. vulnificus* has been reported to be required for serum resistance [33]. This prompted us to examine the impact of *trkH* inactivation on sensitivity to non-decomplemented human serum. The serum had no effect on the viability of the *trkH* mutant and the wild-type strain at all tested concentrations (0–20%) (**Figure 5A**). Hence, in *F. tularensis* LVS, the Trk system is not involved in serum-resistance.

Bacteria respond to variations in the external osmolarity by accumulating or releasing low molecular weight solutes [36]. The most rapid response to osmotic upshift is potassium uptake via specialized transport systems (that in turn triggers other osmotic responses)[37], [38] and the Trk system has been shown to play an important role in this process [36]. A recent study revealed that in the gram-negative symbiotic bacterium *Sinorhizobium melitoti*, the Trk system was the main system involved in accumulation of potassium after an osmotic shock [39]. We therefore monitored (**Figure 5B**) the impact of *trkH* inactivation on the ability of LVS to survive under high osmotic conditions. LVS and *trkH* mutant strains were cultivated in Schaedler K3 medium supplemented with either 0 mM, 200 mM or 400 mM NaCl and cultures were incubated at 37°C. Growth of LVS was not affected in the presence of 400 mM NaCl. For unknown reasons, LVS grew even to higher OD_600_ in the presence of 200 mM NaCl than in normal Schaedler K3 medium. In contrast, growth of the *trkH* mutant was significantly reduced in the presence of 200 mM NaCl and growth was essentially abolished in the presence of 400 mM NaCl. These results suggest a role of TrkH in *F. tularensis* resistance to osmotic shock.

### Impact of the *trkH* Mutation on Bacterial Virulence

**Figure 6:**
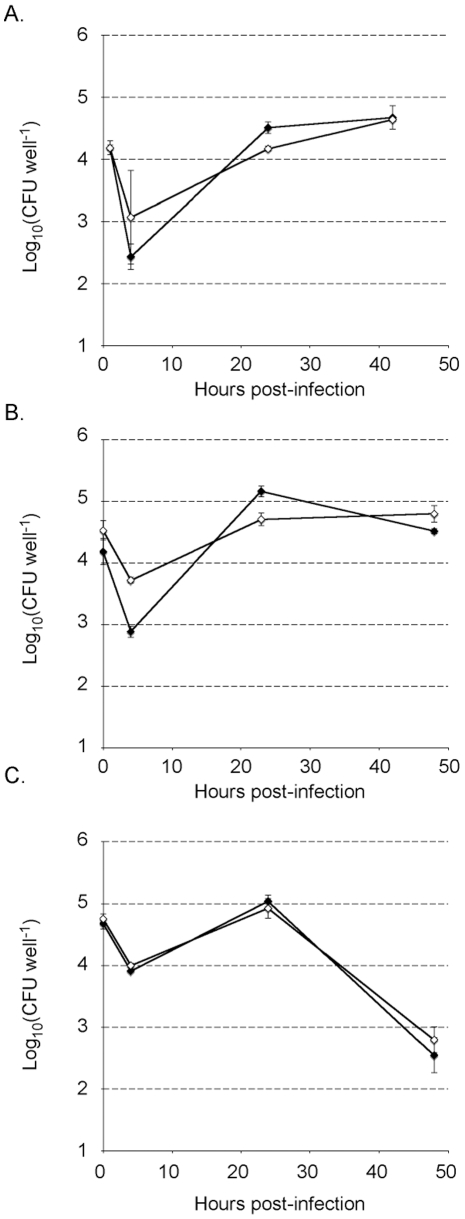
Intracellular replication of LVS and *trkH* strains. Intracellular bacterial replication was monitored over a 48 h-period in J774 macrophage like cells (A), in THP1 human macrophages (B), and in mouse bone marrow-derived macrophages (BMM) from BALB/c mice (C). Results are shown as the average of log_10_ (CFU well^−1^) ± standard deviation. Closed symbols designate the LVS strain and open symbols the *trkH* strain.

To examine if TrkH contributes to *Francisella* pathogenesis, we first compared the ability of *F. tularensis* LVS and the *trkH* mutant to multiply inside macrophages *in vitro.* For this, we infected murine macrophage-like J774 cells, mouse bone marrow-derived macrophages, and human macrophage THP1 cells with the wild type and mutant strains. In all cell types, intracellular growth of the *trkH* mutant was undistinguishable from that of the wild-type strain (**Figure 6**), indicating that *trkH* inactivation has no impact on intracellular survival of LVS.

**Figure 7:**
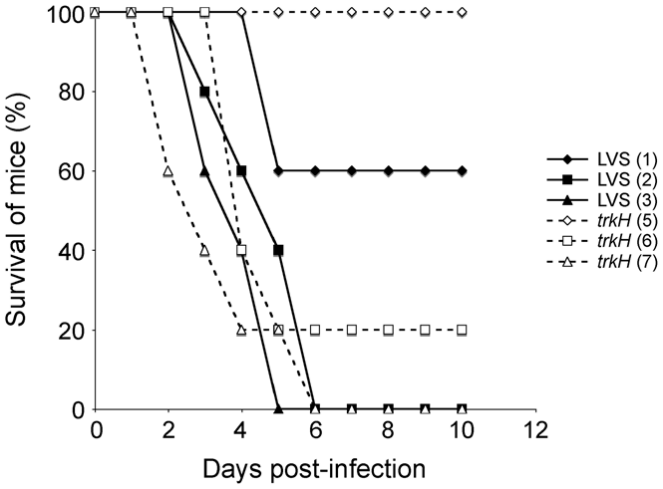
The *trkH* mutant is attenuated for virulence in mice. Groups of five BALB/c mice were infected with LVS (closed symbols) or *trkH* (open symbols) bacteria at different doses by the i.p. route. The log_10_ of the numbers of bacteria used for infection are shown in legend in parenthesis. The survival of mice (in %) was followed for 10 days after infection.

Then, to determine if TrkH is important for the ability of *F. tularensis* to cause disease, we infected 6–8 weeks old BALB/c mice with the LVS and *trkH* strains. Groups of five mice were inoculated by the intraperitoneal (i.p.) route with different numbers of bacteria and survival of the mice was followed for 10 days (**Figure 7**). None of the mice infected with LVS at doses of 100 bacteria survived infection. In contrast, all mice survived infection with *trkH* with doses up to ∼10^5^ bacteria and only infection with a dose of ∼10^6^ *trkH* bacteria or higher resulted in death. These results demonstrate that virulence is severely attenuated in the *trkH* mutant.

**Figure 8:**
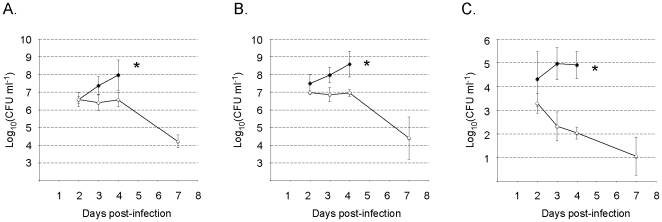
In vivo dissemination of LVS and *trkH.* BALB/c mice were infected with approximately 10^4^ LVS or *trkH* bacteria by the i.p. route. After 2, 3, 4, and 7 days of infection, groups of five mice were sacrificed and the bacterial burden in the spleen, liver, and blood was determined by plating tissue homogenates on chocolate agar. *: all mice infected by LVS died before day 7.

To investigate the fate of the bacteria inside host tissues, we infected mice with ∼10^4^ LVS or *trkH* bacteria and followed the kinetics of infection by assessing the number of viable bacteria in the spleen, liver and whole blood. Groups of five mice were sacrificed 2, 3, 4, and 7 days after infection and viable bacterial numbers were determined by plating of diluted tissue homogenates (**Figure 8A, B**). Blood samples, collected before the mice were sacrificed, were also plated for numerations. LVS increased in numbers from day 2 to day 4 post-infection, reaching ≥10^8^ bacteria per organ (spleen and liver, **Figure 8A, B**) and none of the mice infected with LVS survived the infection to day 7. The *trkH* strain was initially detected in both spleen and liver at identical or slightly lower levels than the wild-type strain. The bacterial numbers then remained high and constant up to day 4 in both organs (10^6^–10^7^ per organ). On day 7, bacterial numbers had significantly decreased but both spleen and liver were still infected (**Figure 8A, B**) and all mice survived the infection. These data indicate that the *trkH* mutant is able to infect mice and to persist for some days in the infected organs. However, it is unable to multiply efficiently inside host tissues and cause disease.

In whole blood, the number of bacteria recorded at day 2 was slightly higher for LVS than for the *trkH* mutant (**Figure 8C**). Strikingly, at day 3, the number of LVS bacteria in blood was increased whereas the number of *trkH* mutant bacteria had drastically diminished. The number of mutant bacteria was even lower the following day (when wild type bacteria had reached a plateau) and *trkH* bacteria were almost absent in the blood at day 7 (**Figure 8C**).

Hence, at days 3 and 4 when only approximately a ten-fold difference in bacterial numbers was observed in the organs of mice infected with either LVS or the *trkH* mutant, a 1,000-fold difference was recorded in the blood.

### Ex Vivo Multiplication in Murine Blood

**Figure 9:**
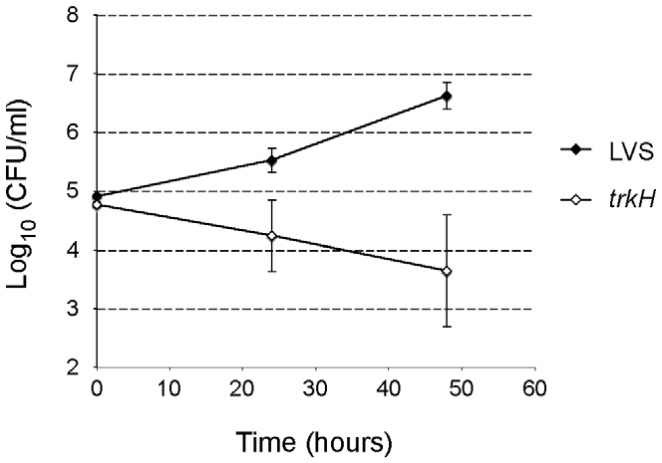
Multiplication of *trkH* mutant is impaired in murine blood ex vivo. Heparinized blood samples from eight mice were inoculated with ∼5×10^4^ bacteria ml^−1^ and incubated at 37°C with shaking for 24 and 48 h, at which point the number of viable bacteria was determined. Closed symbols correspond to the LVS strain and open symbols the *trkH* mutant.

To further examine the apparent decreased capacity of the *trkH* mutant to survive in host blood, we incubated LVS and *trkH* mutant bacteria in murine blood ex vivo. Heparinized blood collected from 8 mice was inoculated with LVS or *trkH* and the viable number of bacteria was determined after 24 and 48 h of incubation at 37°C. As had been observed previously [26], LVS multiplied and increased in numbers after incubation in murine blood (**Figure 9**). In contrast, the viable number of the *trkH* mutant was diminished considerably over the 48 h period, strongly indicating that the mutant fail to multiply in murine blood as the wild type parent strain.

## Discussion

The present work identifies for the first time a gene involved in the extracellular phase of *F. tularensis* infectious cycle and unravels a novel –mineral- nutritional requirement of *F. tularensis*. Our initial aim was to identify novel nutritional requirements involved in *F. tularensis* pathogenesis, by screening banks of *F. tularensis* LVS mutants on chemically defined medium. The unexpected selection of a transposon insertion mutant in a gene encoding a homolog of the potassium permease TrkH, prompted us to address the role of this mineral in *F. tularensis* pathogenesis. We found that the *trkH* mutant required a high level of potassium supplementation to grow in broth. This mutant multiplied normally in cells in culture, but was particularly affected in its ability to survive and multiply in the blood of infected animals.

### Trk Constitutes the Unique Functional Potassium Uptake System of *F. tularensis*

A relatively constant and high intracellular potassium concentration (300 to 500 mM) appears to be essential for bacteria, notably, to maintain cell turgor and to adapt to osmotic variations [36]. For this, bacteria often possess multiple dedicated active potassium transport systems (ion pumps and exchangers) and three major types of transporters have been identified. Trk and Kup are two low affinity potassium transport systems, which are constitutively expressed. The third system is a high affinity system, Kdp, which is normally induced at low potassium concentration. The Trk transporters are widespread in bacteria and in media containing more than 1 mM potassium, Trk is the predominant uptake system. Kup is a potassium uptake system of modest affinity that is found in some bacteria. Kup activity in *E. coli* is more important at low pH where its maximum rate exceeds that of Trk. Finally, Kdp is an inducible P-type ATPase expressed only when the needs for potassium are not satisfied by other transport systems, and thus plays a vital role when the ion is present at concentrations too low to be efficiently taken up by the other potassium transport systems. Transcription of the inducible *kdp* operon is tightly regulated by a two-component system (TCS), composed of the sensor kinase KdpD and the response regulator KdpE [40].

Most of the studies on the role of potassium uptake in bacteria have been carried out in non-pathogenic species. The only two examples describing a role for potassium uptake systems in bacterial pathogenesis concern the intracellular pathogen *S. typhimurium* [41] and the extracellular pathogen *Helicobacter pylori* [42]. *S. typhimurium* seems to possess all three known potassium uptake systems (TrK, Kdp and Kup). In contrast, *H. pylori* lacks all three systems and is likely to take up potassium only via potassium channels. Of note, a recent screen in *Vibrio cholerae* for in vivo inducible genes in humans identified 217 candidate genes, including the *trkH* and *trkA* genes [43]. However, the contribution of *trkH* and *trkA* to bacterial virulence was not addressed. Our genomic analyses suggest that *F. tularensis* subsp. *tularensis* and *holarctica* possess only a Trk-like potassium transporter. The fact that the *trkH* mutant was selected on the basis of impaired growth on solid CDM (which contains *ca*. 18 mM potassium) further supports this notion. The ability of the *trkH* mutant of LVS to grow at high external potassium concentration suggests that enough potassium can cross the bacterial envelope, possibly via hypothetical diffusion channels, to promote survival.

### Importance of Potassium for In Vivo Dissemination of *F. tularensis*

Bacteraemia is a severe complication of a number of infections and can have several dramatic consequences such as sepsis and septic shock, particularly with gram-negative bacteria [44]. A number of pathogenic bacterial species use blood to spread from their initial port of entry through the infected host, and establish infectious foci in specific target organs or tissues [45]. These bacteria have therefore developed a number of defense mechanisms to survive killing in this hostile environment. In addition to defense mechanisms, such as resistance to complement-mediated lysis or resistance to antimicrobial peptides, survival and multiplication in blood requires bacterial adaptation to this peculiar nutritional environment. However, only limited information is currently available on the nature of bacterial requirements in blood. For example, iron is known to be an essential ion for the majority of bacterial species. The selection and analysis of auxotrophic mutants have also highlighted the role of amino acid and nucleic acid uptake and biosynthesis in the virulence of a number of pathogenic bacteria. However, the specific requirement of these systems for blood stage dissemination is not established. Interestingly, a recent study [30] has demonstrated that de novo synthesis of purines and pyrimidines was required for survival of *E. coli* in human blood. Furthermore, inactivation of nucleotide biosynthesis genes in *Bacillus anthracis* and *S. typhimurium* also prevented growth in human serum, strongly suggesting that nucleotide precursors are limiting for bacterial growth in blood.

Remarkably, while the potassium concentration is higher than 100 mM inside human cells, the extracellular concentration in mammalian hosts are usually less than 10 mM [46], and the normal concentration of potassium in human blood serum is only 3.5 to 5.0 mM [47]. Hence, it is reasonable to assume that adaptation to variations in potassium concentration must play an important role in the pathogenicity of facultative intracellular pathogens, such as *F. tularensis*, which are also found free in the bloodstream during systemic dissemination. Although the role of potassium homeostasy in the physiology of eukaryotic organisms has been extensively studied [48], the role of potassium acquisition systems in the virulence of human pathogens has not been well characterized [41], [42], [49].

We used here a simple screen on chemically defined medium (CDM) to search for potentially new nutritional requirements of *F. tularensis*. This approach allowed us to select a mutant in a gene encoding the predicted potassium permease TrkH of the Trk uptake system. Remarkably, the *trkH* gene had never been identified in previous screens. While the mutant grew only poorly in CDM (containing 18 mM potassium), normal growth of the *trkH* mutant was restored by adding potassium at high concentration, establishing the importance of TrkH in potassium acquisition. Furthermore, as would be expected of a mutant in the potassium transporter, growth of the *trkH* mutant was also significantly reduced at high osmolarity. In contrast, inactivation of *trkH* had only marginal impact on the capacity of the bacterium to multiply intracellularly in mouse or human cell lines and in primary bone marrow macrophages. This result is not surprising given the high potassium content (100–300 mM range) of eukaryotic cells. However, the virulence of the trkH mutant was markedly attenuated. In vivo kinetics of bacterial multiplication revealed a rapid elimination of the *trkH* mutant, particularly in the blood of infected mice. The specific growth defect of the *trkH* mutant was further confirmed in blood ex vivo. LVS multiplied after incubation in murine whole blood [26] while the *trkH* mutant failed to multiply.

At any rate, it cannot be excluded that the impaired survival in CDM and virulence defect of the *trkH* mutant might not be totally attributable to its potassium uptake deficiency. It could be due, for example, to another unreported function of the protein or to another spontaneous mutation or to some polar effects.

It has been previously shown that in bacteremic mice, bacteria were mainly extracellular and that moreover, both *F. tularensis* LVS and Schu S4 failed to grow in murine plasma [26]. We also found that LVS (and the *trkH* mutant) was not able to multiply in mouse serum (not shown), suggesting that they require host cells or cellular component to replicate in blood. We are currently evaluating the transcriptomic response of *F. tularensis* LVS in mouse serum to further understand how the bacterium adapts to this peculiar nutritional environment.

In conclusion, the potassium requirement specific to the blood stage is in support of the importance of the extracellular phase in *F. tularensis* pathogenesis. More generally, active potassium uptake systems are likely to be important for all bacterial pathogens able to disseminate in vivo extracellularly via the hematogenous route. Specific inhibition of TrkH could lead to the development of potential new therapeutic strategy against *F. tularensis,* which might be applicable to other bacteria with systemic dissemination.

## Materials and Methods

### Strains, Media and Chemicals

*F. tularensis* LVS strain was obtained from Dr Anders Sjöstedt (Umeå University, Sweden), and grown at 37°C in Schaedler K3 broth (Biomerieux SA Marcy l'Etoile, France), Chamberlain defined synthetic medium [35] or chocolate agar enriched with PolyViteX (chocolate agar) (Biomerieux). *E. coli* was grown in Luria-Bertani (LB) medium at 37°C. When required, the medium was supplemented with kanamycin (10 µg ml^−1^). Kanamycin was purchased from Sigma-Aldrich (St Louis, MO). Oligonucleotide primers were synthesized by Eurogentec (France). Primers and bacterial strains used in this study are listed in **Table 2**.

**Table 2:** Bacterial strains, plasmids, and oligonucleotides used in study.

Strain, plasmid, or primer		Description	Reference
*E. coli*			
	DH5α λ*pir*	F− φ80*lacZ*ΔM15 *endA1 recA1 hsdR17 supE44 thi-1 gyrA96 relA1* Δ(*lacZYA-argF)U169,* lysogenized with λ*pir* phage; used a host for *HimarFT* rescue	Laboratory strain collection
*F. tularensis*			
	LVS	*F. tularensis* subspecies *holarctica* live vaccine strain	Laboratory strain collection
	LVS *trkH*::*HimarFT*	LVS containing a *HimarFT* insertion in *trkH*	This study
Plasmids			
	pFNLTP16 H3	*E. coli-F. tularensis* temperature sensitive shuttle vector containing *HimarFT*, Km^r^ Ap^r^	[31]
	pHimar 3	4.3 kb derivative of pFNLTP16 H3 lacking *F. tularensis* replicon (*repA)*, Km^r^	This study and [22]
	pFNLTP6*gro*	Shuttle vector with *F. tularensis groEL* promoter, Km^r^ Ap^r^	[31]
	p6Tc-*gro*	Derivative of pFNLTP6*gro,* Tc^r^	This study
	p6Tc-*gro-trkH*	p6Tc-*gro* with fragment harboring *trkH* and upstream and downstream regions cloned in *Eco*RI site, Tc^r^	This study
Primers			
	K1	5′-GCTATTCGGCTATGACTG-3′, forward primer for kanamycin marker (*npt*) probe	[31]
	K2	5′-CAGCAATATCACGGGTAG-3′, reverse primer for kanamycin marker (*npt*) probe	[31]
	K3	5′-GCTTCCTCGTGCTTTACGG-3′, *npt* primer for *HimarFT* insertion sequencing	[31]
	R1	5′-TGCCACCTAAATTGTAAGCG-3′, R6K primer for *HimarFT* insertion sequencing	[31]
	E1	5′-CCGGAATTCGGAAACTAGCTTTAGGCTTTG-3′, forward primer for complementation	
	E3	5′-CCGGAATTCCGATTACAAAGTTATCGCATATG-3′, reverse primer for complementation	

### Generation of Banks of LVS Mutants and Screening Procedure

We used the modified pHimar3 suicide mutagenesis system [22], derived from the temperature sensitive plasmid pFNLTP16 H3, to generate *F. tularensis* mutant libraries [31]. As described previously, the pFNLTP16 part of the plasmid was removed by digestion with *Not*I followed by ligation. The resulting 4.3 kb pHimar3 plasmid (containing the *HimarFT* transposon, the transposase gene, the neomycin phosphotransferase gene under the control of *Francisella groEL* promoter and the *E. coli*-compatible origin of replication *oriR6K*) was transformed into *E.coli* DH5*αλ pir*, and selection of transformants was performed on LB agar containing 50 µg ml^−1^ of kanamycin. Transposition of the *HimarFT* transposon was performed by electroporation of 100–200 ng of plasmid pHimar3 into electrocompetent LVS. Electroporation was performed as described previously [24]. Transformed cells were grown in Schaedler K3 for 3 h at 37°C with shaking and then spread onto chocolate agar plates enriched with BD BBL™IsoVitaleX (Becton Dickson) and containing 10 µg ml^−1^ of kanamycin. Plates were incubated at 37°C for 3–4 days to recover individual clones containing single *HimarFT* chromosomal insertion.

To isolate auxotrophic *Francisella* LVS mutants, we screened a library of 2,500 mutants for growth on CDM agar plates. For this, individual colonies were simultaneously streaked onto chocolate agar and CDM plates. A series of mutants which were unable to grow on CDM after 24 h of incubation at 37°C were selected and further analyzed by Southern blot as described previously [24]. Southern blot analysis performed on the two *trkH* mutants and on the 4 auxotroph mutants, confirmed that they (**Table 2**) contained a single chromosomal transposon insertion (**Figure S1**).

### Determination of the Insertion Site

Transposon insertion sites were mapped as described previously [24]. Briefly, genomic DNA was isolated from each mutant strain and 1 µg was digested with *Spe*I overnight and subsequently ligated with T4 DNA ligase. *Himar*-containing fragments were recovered as plasmids in *E. coli* DH5α λpir and the transposon insertion site was determined by sequencing. The genomic location of each transposon insertion is listed in **Table 1**.

### Cell Cultures and Macrophage Infection

J774 (ATCC Number: TIB67) and THP1 (ATCC Number: TIB-202™) cells were propagated in RPMI or Dulbecco's Modified Eagle's Medium (DMEM) containing 10% fetal calf serum. Cells were seeded at a concentration of ∼2×10^5^ cells per well in 12-well cell tissue plates and monolayers were used 24 h after seeding. Bone marrow-derived macrophages (BMM) from BALB/c mice were obtained and cultured as described [24]. J774 and THP1 macrophage monolayers were incubated for 90 min and BMM for 60 min at 37°C with the bacterial suspensions (approximate multiplicities of infection 100) to allow the bacteria to enter. After washing (time zero of the kinetic analysis), the cells were incubated in fresh culture medium containing gentamicin (10 µg ml^−1^) to kill extracellular bacteria. At several time-points, cells were washed three times in RPMI or PBS, macophages were lysed by addition of water and the titer of viable bacteria released from the cells was determined by spreading preparations on agar plates. For each strain and time in an experiment, the assay was performed in triplicate. Each experiment was independently repeated at least two times and the data presented corresponds to one typical experiment.

### Infection of Mice and Virulence Assays

Animal experiments were approved by the Institut National de la Santé et de la Recherche Médicale (INSERM) and performed according to the guidelines of the INSERM for laboratory animal husbandry.

Bacteria were grown in Schaedler K3 medium to an OD_600_ of 0.3–0.4 and then diluted to the appropriate concentration (CFU/ml) in 0.15M NaCl. 6- to 8-week-old female BALB/c mice (Janvier, Le Genest St Isle, France) were i.p. inoculated with 200 µl of bacterial suspension. Groups of five mice were inoculated with various doses of bacteria and the mortality was followed for 10 days. The actual number of viable bacteria was determined by plating dilutions of the bacterial suspension on chocolate agar.

For experiments following the kinetics of infection, BALB/c mice were infected with ∼10^4^ CFU of LVS or *trkH* mutant bacteria. At 2, 3, 4, and 7 days after infection, groups of five mice were sacrificed and the bacterial number was determined in the liver, spleen and blood by plating homogenized samples on chocolate agar plates.

### Functional Complementation

First, the plasmid p6Tc-*gro* was constructed by substituting a *Pvu*I-*Pst*I fragment of pFNLTP6*gro* with the *tet* gene amplified by PCR from plasmid pACYC184. Then, the *trkH* gene and upstream region was amplified by PCR using primers E1 and E3 and cloned in the *Eco*RI site of p6Tc-*gro*, yielding recombinant plasmid pC-trkH. The sequence of *trkH* was verified by sequencing. The recombinant plasmid pC-*trkH* was introduced in the *trkH* mutant strain by electroporation.

### Sensitivity to Human Serum

The sensitivity assay was performed essentially as described previously [50]. Briefly, bacteria (5×10^5^) from mid-exponential phase culture were suspended in 0.5 ml of RPMI containing 20 mM HEPES (N-2-hydroxyethylpiperazine-N'-2-ethanesulfonic acid) buffer (PAA laboratories GmbH). The suspensions were incubated at 37°C with shaking in the presence of increasing concentration of non-decomplemented human serum (0–20%) (PAA Laboratories, GmbH Pasching). Serial dilutions in RPMI-HEPES were plated on chocolate agar plates to determine the number of viable bacteria at the beginning and after 1 h of incubation.

### Kinetics of Growth and Stress Resistance

We studied the growth kinetics of the two strains in CDMx containing increasing concentrations of potassium in the form of KCl (0, 10 µM, 100 µM, 1 mM, 10 mM and 100 mM). The KH_2_PO_4_ and K_2_HPO_4_ present in normal CDM were substituted by NaH_2_PO_4_ and Na_2_HPO_4_ to maintain the proper pH.

To test sensitivity to osmotic shock, LVS and the *trkH* strains were grown in the absence or in the presence of high concentration of NaCl. Bacteria were grown overnight in Schaedler K3 medium and then diluted 10-fold in fresh medium containing either 200 mM or 400 mM NaCl and incubated at 37°C with agitation. Growth was assessed by measuring the optical density at 600 nm.

### Growth of LVS and TrkH Mutant in Murine Blood

Animal experiments were performed according to the guidelines of the Institut National de la Santé et de la Recherche Médicale (INSERM) for laboratory animal husbandry.

Terminal blood was collected from eight 6– to 8-week-old female BALB/c mice (Janvier, Le Genest St Isle, France) using heparin as an anticoagulant (200 UI of heparin ml^−1^). Each blood sample was divided in 4 fractions in sterile 2 ml tubes. Two fractions of each sample were inoculated with ∼5×10^4^ bacteria (10 µl in 0.15M NaCl solution) of either LVS or *trkH* mutant. Fractions were incubated at 37°C with agitation (120 rpm). The number of viable bacteria was determined at 24 h and 48 h by plating serial dilutions on chocolate agar.

### Bioinformatics Analyses

LVS genome sequence analyses were performed essentially from the KEGG database (http://www.genome.jp/kegg-bin/show_organism?org=ftl) and associated sofware.

*F. tularensis* complete genomes were available at the NCBI entrez Genome project site (http://www.ncbi.nlm.nih.gov/sites/entrez?db=genomeprj&cmd=Retrieve&list_uids=20197,16421,17265,19571,17375,9,18459).

The program BLASTN 2.2.22 was used on the nucleotide collection (nr/nt) available at http://www.ncbi.nlm.nih.gov/blast/Blast.cgi?PROGRAM=blastn&BLAST_PROGRAMS=megaBlast&PAGE_TYPE=BlastSearch&SHOW_DEFAULTS=on&LINK_LOC=blasthome.

Protein sequence similarity searches were performed using BLASTP [51] against non-redundant protein data bases. Multiple sequence alignment of homologous sequences was carried out using ClustalW [52].TMHMM server version 2.0 (http://www.cbs.dtu.dk/services/TMHMM-2.0/) was used to perform TMS predictions.”

## Supporting Information

Figure S1 Southern blot analysis. Genomic DNA (5 µg) of six transposon insertion mutants was digested with SpeI overnight, resolved on a 0.7% agarose gel and transferred to a positively charged nylon membrane (Amersham Pharmacia, RPN203B) by capillary action for 4 h. Blots were probed with DNA fragments randomly labeled using random prime labelling system kit (Rediprime™ II, Amersham pharmacia, RPN16330l/AE), for which a 634-bp fragment corresponding to the neomycin phosphotransferase (npt) gene was used as probe. pHimar3.4, SmaI-linearized plasmid pFNLTP16 H3; trkH-clone 1, transposon insertion at nucleotide 216 of trkH coding sequence; trkH-clone 2, transposon insertion at nucleotide 816 of trkH coding sequence; trpE, transposon insertion in gene FTL_1966 ; aroE, transposon insertion in gene FTL_0173; purL, transposon insertion in gene FTL_1860 ; purF, transposon insertion in gene FTL_1861.
(1.26 MB TIF)
