# Supplementary figures and images for: Identification of trkH, Encoding a Potassium Uptake Protein Required for Francisella tularensis Systemic Dissemination in Mice
Source: PLoS One. 2010 Jan 29;5(1):e8966. doi: 10.1371/journal.pone.0008966 (PMC2813290; doi:10.1371/journal.pone.0008966)

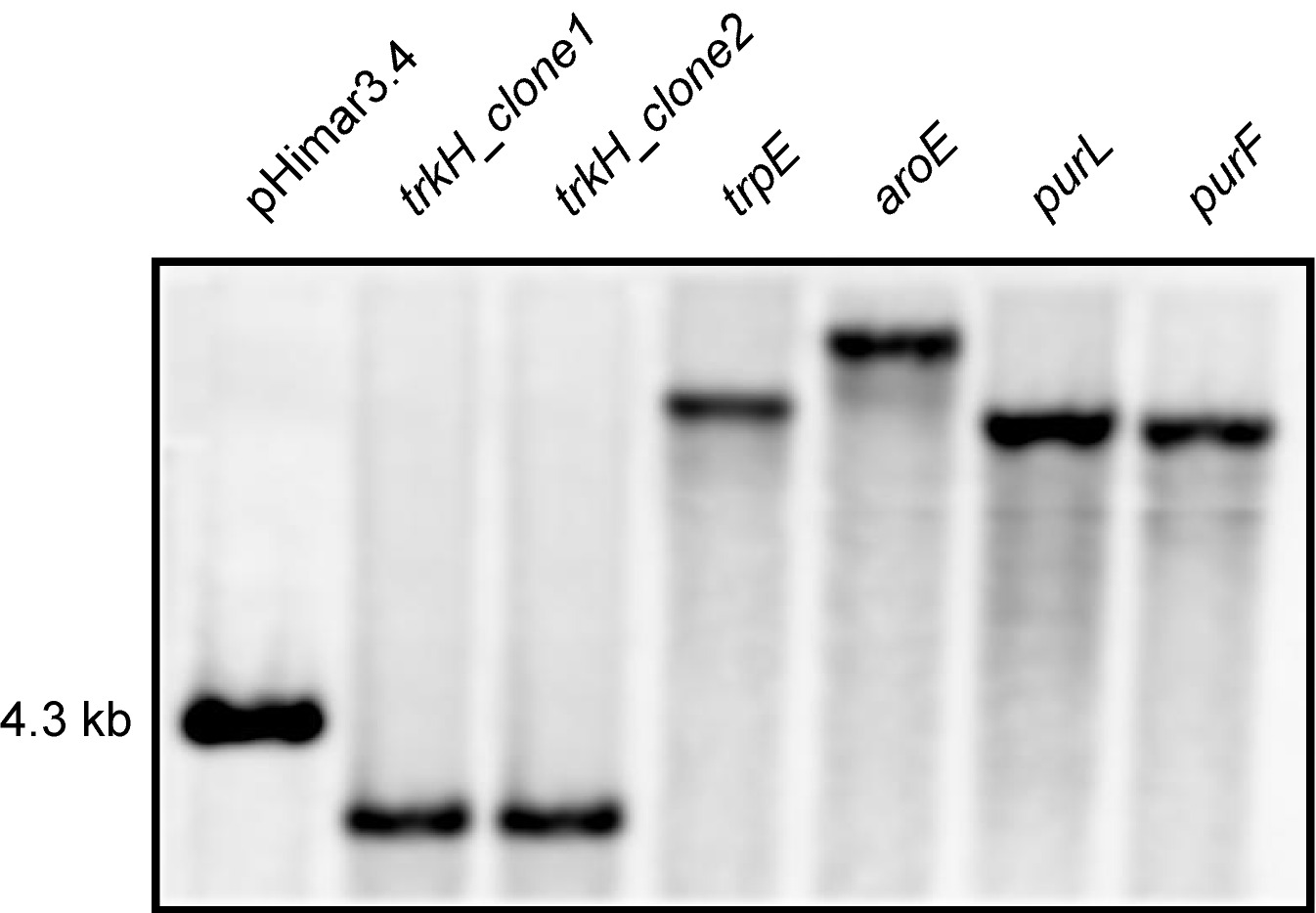

Supplement: Figure S1 — Southern blot analysis. Genomic DNA (5 µg) of six transposon insertion mutants was digested with SpeI overnight, resolved on a 0.7% agarose gel and transferred to a positively charged nylon membrane (Amersham Pharmacia, RPN203B) by capillary action for 4 h. Blots were probed with DNA fragments randomly labeled using random prime labelling system kit (Rediprime™ II, Amersham pharmacia, RPN16330l/AE), for which a 634-bp fragment corresponding to the neomycin phosphotransferase (npt) gene was used as probe. pHimar3.4, SmaI-linearized plasmid pFNLTP16 H3; trkH-clone 1, transposon insertion at nucleotide 216 of trkH coding sequence; trkH-clone 2, transposon insertion at nucleotide 816 of trkH coding sequence; trpE, transposon insertion in gene FTL_1966 ; aroE, transposon insertion in gene FTL_0173; purL, transposon insertion in gene FTL_1860 ; purF, transposon insertion in gene FTL_1861. (1.26 MB TIF) [file pone.0008966.s001.tif]
